# Low Interest Among Young People in Becoming Nurses in Greece: Contributing Factors According to Academic Staff

**DOI:** 10.3390/nursrep16020049

**Published:** 2026-01-30

**Authors:** Petros Galanis, Ioannis Moisoglou, Christos Triantafyllou, Joao Breda, Pavlos Myrianthefs

**Affiliations:** 1 Faculty of Nursing, National and Kapodistrian University of Athens, 11527 Athens, Greece; pegalan@nurs.uoa.gr (P.G.); pmiriant@nurs.uoa.gr (P.M.); 2 Department of Nursing, University of Thessaly, 41500 Larissa, Greece; iomoysoglou@uth.gr; 3 WHO Athens Quality of Care and Patient Safety Office, 10675 Athens, Greece; rodriguesdasilvabred@who.int

**Keywords:** nursing, nurses, academic staff, baccalaureate programs, motives, profession

## Abstract

**Background:** The nursing profession is currently facing a critical challenge with a noticeable decline in interest among young people to pursue nursing as a career. **Objectives:** This study examined academics’ perceptions of factors driving low enrollment in Greek baccalaureate nursing programs and explored incentives that could motivate young people to pursue nursing careers. **Methods:** We performed a cross-sectional study. We collected our data during October 2025 through an anonymous questionnaire. Source population included all academics in the nine nursing departments in Greece. Response rate was 54.2% (90 out of 166). **Results:** We classified the factors contributing to the low interest in baccalaureate nursing education programs into four groups: (a) poor working conditions, (b) negative social and cultural perceptions, (c) educational constraints, and (d) impact of the COVID-19 pandemic. Academics identified negative social and cultural perceptions of nursing and poor working conditions as the primary drivers of low interest in baccalaureate nursing programs. The COVID-19 pandemic was viewed as having a moderate influence on young people’s career choices, while educational constraints were considered least important overall. Academics in nursing departments based in Greece’s capital perceived the pandemic’s impact as more substantial than colleagues outside the capital and attributed greater importance to educational constraints. Respondents without prior clinical nursing experience emphasized educational barriers more strongly. To attract students, academics prioritized improving working conditions, increasing salaries, and expanding scholarships and support. **Conclusions:** Academics reported that unfavorable nursing work environments, intensified during COVID-19, influence students’ career choices, underscoring the need for urgent policy and organizational actions informed by this study and existing evidence.

## 1. Introduction

Nurses constitute the largest professional group within healthcare organizations, representing the backbone of service delivery across care settings. Their contribution extends beyond the volume of care delivered, as they play a decisive role in determining the overall quality and safety of healthcare services. Evidence consistently demonstrates that patient satisfaction and safety during hospitalization are closely associated with the quality of nursing care [1,2]. Ensuring optimal care delivery is fundamentally dependent on adequate nurse staffing. Patients admitted to understaffed units face a substantially higher risk of mortality, readmission, longer length of stay, and adverse events, such as pressure injuries and healthcare-associated infections, compared with those in adequately staffed units [3,4,5]. Persistent nurse understaffing represents a longstanding and systemic challenge for healthcare organizations worldwide, rendering it the Achilles’ heel of contemporary health systems.

Nursing understaffing, although ostensibly shaped by organizational decisions regarding recruitment, is fundamentally driven by two critical underlying factors. The first concerns the attrition of nurses who exit the profession, while the second, which poses an even greater long-term threat of “desertification” of the nursing workforce, is the declining interest of students in choosing nursing as a career. These two drivers are interrelated. Focusing first on nurse attrition, a substantial range of factors associated with the nursing work environment can influence the decision to leave the profession. Work environment determinants such as high job demands, excessive workload, overtime obligations, inadequate financial compensation, lack of leadership and peer support, limited career prospects, the poor public image of nursing, and occupational burnout collectively constitute the internationally recognized set of contributors that compel nurses to leave the profession [6,7,8,9,10,11]. In a recent study conducted in the United States among nurses with 30 years of professional experience who ended their health care employment, two of the four primary reasons identified were burnout or emotional exhaustion and insufficient staffing (the other two were planned retirement and family obligations) [12]. This highlights a vicious cycle in which nursing personnel, and consequently healthcare organizations, are trapped: poor working conditions and understaffing lead to nurse burnout [13,14,15]; burnout leads to turnover, and turnover further exacerbates understaffing [6,16]. Importantly, experienced and highly skilled nursing staff cannot be readily replaced, as replacement is not merely numerical; the qualitative attributes and expertise of those departing must also be matched. According to the World Health Organization, although global shortages of nursing personnel have shown a declining trend, it is nevertheless estimated that by 2030 there will be a deficit of 4.1 million nurses [17].

The COVID-19 pandemic, characterized by the large-scale admission of critically ill patients, created exceptionally demanding working conditions, with nurses experiencing burnout, moral distress, increased workload with extensive overtime, insufficient resources and support, and the fear of infecting their family or friends and the fear of the death of a family member or friend [18,19,20]. In addition, they were found to experience a range of mental health problems, including insomnia, anxiety, depression, post-traumatic stress disorder (PTSD), and elevated stress levels [21]. In the already highly demanding working conditions faced by nurses, the COVID-19 pandemic added substantial workload and occupational strain, triggering a marked exodus from the profession and a widespread intention to leave. In the United States alone, during the pandemic approximately 100,000 registered nurses left the workforce due to stress, burnout, and retirement, while an additional 610,388 registered nurses reported an intent to leave the workforce by 2027 for similar reasons. Comparable trends have been observed among younger nurses, whose intention to leave has also risen sharply [22]. Studies among nursing personnel worldwide have consistently revealed very high rates of reported intention to leave [23,24]. Given that intention to leave is a well-established predictor of actual turnover [25], the outlook regarding the further contraction of the nursing workforce appears particularly alarming. Studies conducted in Greece in post COVID-19 era indicate that 40–56% of nursing staff report a turnover intention [26,27], at a time when the country is experiencing severe understaffing and ranks among the lowest OECD countries in terms of the number of nurses per 1000 inhabitants [28].

The second critical factor contributing to the nursing shortage is the declining willingness of students to pursue nursing studies. This factor is particularly significant, as nursing students constitute the pool of the future workforce, one that appears to be gradually diminishing. According to findings from the Programme for International Student Assessment (PISA), the proportion of 15-year-old students who express interest in pursuing a nursing career has declined in at least half of OECD member states between 2018 and 2022. On average, the percentage of adolescents intending to become nurses decreased from 2.3% in 2018 to 2.1% in 2022 across OECD countries. This downward trend is especially pronounced in the United States, Canada, several Nordic countries (including Norway and Denmark), Ireland, the United Kingdom, and Switzerland [29]. With regard to Greece, the proportion of students expressing interest is exceptionally low, reaching approximately 1%, about half the average reported across OECD countries, and shows a slight decline in 2022 compared with 2018 [29]. The 2025 data for Greece regarding students’ admissions to baccalaureate nursing education indicate that, out of 1400 available positions, 400 remained unfilled (corresponding to 28.6%). During the pandemic, although the work of nurses was internationally acknowledged and they were widely portrayed as heroes, this positive public image was insufficient to motivate students to pursue nursing studies. In weighing their decision regarding enrollment in nursing programs, the profession’s favorable portrayal ultimately could not outweigh the demanding working conditions and dissatisfaction with financial remuneration. The PISA study recommends that countries address these two factors, as they constitute critical policy interventions for attracting young people to the nursing profession [29]. Other factors that may act as barriers to students’ admission to baccalaureate nursing education include geographical location, ethnic characteristics, the perception of nursing as a low-status job [30,31], and, more recently in the United States, the non-recognition of nursing as a professional, which will exclude nursing students from access to student loans and consequently lead either to dropping out of their studies or to not choosing nursing in the first place [32].

The nursing profession in Greece is currently facing a critical challenge: a noticeable decline in interest among younger generations to pursue nursing as a career. This trend has significant implications for the healthcare system, which relies heavily on a steady influx of qualified professionals to meet growing patient care demands. Despite the increasing need for nurses due to demographic changes and evolving healthcare needs, nursing programs struggle to attract and retain students. At the same time, an aversion among newly graduated nurses to working in hospitals is also being observed. Younger nurses, being aware of the challenging working conditions in hospital settings (such as understaffing and frequent night shifts), tend to opt either for school nursing positions or for employment within primary healthcare structures. In fact, they often resign from hospital posts and transition to these settings. More experienced nurses, however, are also leaving hospital roles, similarly choosing to pursue the specialty of school nursing. Although official data regarding these workforce shifts are not yet available, the Greek Minister of Health has recently highlighted this phenomenon.

It is estimated that, globally, there will be a shortage of approximately 4.5 million nurses by 2030, with the most pronounced deficits observed in countries in Africa, South East Asia, and the WHO Eastern Mediterranean Region, as well as in parts of Latin America [33]. Data from the United States and Europe indicate a decline, comparing 2022 with 2018, in the proportion of students choosing to pursue nursing studies [29]. This trend, combined with the substantial number of actively practicing nurses leaving the profession, poses a major challenge for health systems, which are expected to face increasing difficulties in recruiting and retaining adequate nursing personnel.

Social Cognitive Career Theory (SCCT) serves as one of the leading frameworks for understanding how individuals develop educational and career interests, make occupational choices, and perform in their chosen fields. It draws on (a) cognitive-personal factors, such as social perceptions, self-efficacy beliefs, expectations about outcomes, and personal goals, and (b) contextual and experiential influences, including gender, socioeconomic background, academic capability, working conditions, educational constraints, and other environmental elements. The theory emphasizes the need to consider both personal characteristics and contextual conditions to fully explain career decision-making [34]. Evidence supports the applicability and effectiveness of the SCCT across diverse cultural settings [35,36], and therefore we relied on this theory to conceptualize factors that contribute to the low interest in baccalaureate nursing education programs in our study. Moreover, we considered in our study the consequences of the COVID-19 pandemic in healthcare services and especially in the nursing profession since nurses during the pandemic experienced elevated levels of mental health issues such as anxiety, depression, and sleep disturbances [37]. Moreover, nurses during the pandemic experienced high levels of job burnout and low levels of job satisfaction [19]. Therefore, we considered that the COVID-19 pandemic may affect young people’s opinion about nursing profession.

In this context, we considered that factors that contribute to the low interest in baccalaureate nursing education programs could be classified into four categories: poor working conditions, negative social and cultural perceptions, educational constraints, and impact of the COVID-19 pandemic.

Understanding the underlying factors contributing to this lack of engagement among the youth is essential for developing effective strategies to reverse the trend and ensure the sustainability of the nursing workforce. To the best of our knowledge, the opinion of academics regarding the factors that deter young people from entering baccalaureate nursing education programs is unknown. Thus, we examined the factors influencing the inadequate uptake of nursing programs in Greek universities, based on academic perspectives. Additionally, we investigated possible incentives for attracting young individuals to nursing programs, as perceived by academics.

## 2. Materials and Methods

### 2.1. Study Aim

The aim of our study was to examine the perceptions of academics regarding the factors that deter young people from entering baccalaureate nursing education programs. Moreover, we examined possible incentives for attracting young individuals to nursing programs, as perceived by academics.

### 2.2. Study Design

We performed a cross-sectional study in Greece. We collected our data during October 2025 through an anonymous questionnaire. In particular, we used Google forms to create an online version of the study questionnaire. We informed presidents of the nursing departments about the aim and design of the study, and, then, they sent the online version of the study questionnaire to the academic staff through emails. Four courteous reminders were sent throughout a month. There are nine nursing departments in Greece including 166 academics in total. More than half of the academics participated in this study. In particular, the response rate was 54.2% (90 out of 166). The response rate among departments ranged from 36.4% to 100.0%. Two nursing departments are located in the capital of the country (National and Kapodistrian University of Athens and University of West Attica), while the remaining seven are in other cities (i.e., Thessaloniki, Patras, Heraklion, Larissa, Ioannina, Tripoli, Alexandroupoli). This study adhered to the Strengthening the Reporting of Observational Studies in Epidemiology (STROBE) guidelines for cross-sectional studies [38].

### 2.3. Measurements

We measured the following demographic characteristics of academic staff: gender (males or females), age (continuous variable), rank (lecturer, assistant professor, associate professor or professor), work experience as academic (continuous variable), work experience as clinical nurse (no or yes).

We classified the factors contributing to the low interest in baccalaureate nursing education programs into four groups: (a) poor working conditions, (b) negative social and cultural perceptions, (c) educational constraints, and (d) impact of the COVID-19 pandemic. There are no studies measuring the opinion of academics regarding the factors that deter young people from entering baccalaureate nursing education programs. Therefore, there is an absence of questionnaires to measure these opinions. In this context, we reviewed similar literature on this research field to create a pool of potential factors that could influence individuals’ decision to become nurses. For instance, we reviewed studies that examined (a) career-choice motives, nursing orientations and vocational decisions among nursing students [39,40,41], (b) recruitment and retention strategies among nurses [42], (c) the influence of significance others on school pupils’ choice of nursing as a profession [43], (d) the reasons for the decreased interest of school pupils in the nursing profession [44,45].

Afterall, we provided respondents with 17 potential factors (Supplementary Table S1) for the insufficient uptake of baccalaureate nursing education programs in Greece, and we asked them to rate the importance of these factors on a five-point Likert scale from 1 (not at all important) to 5 (extremely important). These 17 factors were classified into four groups (a) poor working conditions (four items), (b) negative social and cultural perceptions (five items), (c) educational constraints (six items), and (d) impact of the COVID-19 pandemic (two items). We calculated a total mean score for the four groups of factors that we described above. For instance, we calculated a mean score for poor working conditions by adding answers in items #1, #2, #3 and #4 (Supplementary Table S1) and dividing them by four. Thus, scores across the four factors ranged from 1 to 5, with higher values indicating greater perceived importance. Cronbach’s alpha for the factors “poor working conditions”, “negative social and cultural perceptions”, “educational constraints” and “impact of the COVID-19 pandemic” were 0.704, 0.864, 0.790 and 0.764, respectively. Therefore, the consistency of the questionnaire was acceptable. Finally, we provided respondents with an open-ended question to further add factors if they want.

Additionally, we provided respondents with 10 potential motives (Supplementary Table S2) that could help attract young people to choose nursing as a profession. Answers were on a five-point Likert scale from 1 (not at all important) to 5 (extremely important). Thus, higher scores indicated greater importance of the motives. Cronbach’s alpha for the motives was 0.852 indicating very good internal reliability. Moreover, respondents had the option to report additional motives by answering an open-ended question.

Prior to administering the study questionnaire, we conducted cognitive interviews with five academic staff members to assess the face validity of the instrument. These interviews were undertaken to ensure that all items were clearly understood, appropriately worded, and interpreted as intended by individuals familiar with the academic and professional context of nursing education. The participants reported that all questionnaire items were comprehensible and aligned with their expected meaning. Consequently, no modifications were deemed necessary, and the questionnaire was retained in its original form for use in the main study.

In brief, our data collection instrument included two sections: (a) factors contributing to the low interest in baccalaureate nursing education programs based on the perspectives of academic staff in nursing departments (17 items), (Supplementary Table S1), and (b) motives for attracting young individuals to nursing programs, as perceived by academics (10 items), (Supplementary Table S2).

### 2.4. Ethical Issues

The study protocol was approved by the Ethics Committee of the Faculty of Nursing, National and Kapodistrian University of Athens (approval number; 02, 16 September 2025). Moreover, we conducted our study in accordance with the Declaration of Helsinki [46]. In particular, we collected our data on an anonymous and voluntary basis. We informed respondents about the aim and the design of our study, and they gave their informed consent.

### 2.5. Statistical Analysis

We present categorical variables as numbers and percentages. Also, we use mean, standard deviation (SD), median and interquartile range to present continuous variables. The Kolmogorov–Smirnov test and Q-Q plots showed that continuous variables (except work experience as academic) followed the normal distribution. We explored associations between demographic characteristics and academics’ views on factors contributing to the low interest in baccalaureate nursing education programs by performing the following statistical tests: (a) independent samples t-test to examine the association between factors and work experience as clinical nurse and academics in nursing departments located in the capital of the country, (b) analysis of variance to examine the association between factors and rank, (c) Pearson’s correlation coefficient to examine the correlation between factors and age since age follow the normal distribution, (d) Spearman’s correlation coefficient to examine the correlation between factors and work experience as academic since work experience did not follow the normal distribution. A *p*-value below 0.05 was regarded as statistically significant. We used the IBM SPSS 28.0 (IBM Corp. Released 2021. IBM SPSS Statistics for Windows, Version 28.0. Armonk, NY, USA: IBM Corp) for the analysis.

## 3. Results

### 3.1. Demographic Characteristics

Table 1 shows demographic characteristics of the study sample. Our study sample included 90 academics. Among them, 52.2% were females and 47.8% were males. Mean age was 53.2 years (SD; 7.5), with a median of 54 years, a minimum age of 32 years and a maximum age of 67 years.

Among our respondents, 34.4% were professors, 26.7% were assistant professors, 23.3% were associate professors and 15.6% were lecturers. Six out of ten academics (64.4%) have worked as clinical nurses. Mean years of work experience as an academic was 12.4 (SD; 8.9), while the median was 12.5 (range; 1 to 34).

### 3.2. Contributing Factors

Table 2 shows descriptive statistics for the factors contributing to the low interest in baccalaureate nursing education programs based on the perspectives of academic staff. Academics considered negative social and cultural perceptions towards nursing profession (mean; 3.86) and poor working conditions (mean; 3.79) as the most important factors. Among the nine items including in these two factors, the most important issues were poor work environment (mean; 4.53), low salaries (mean; 4.27), lack of social recognition (mean; 4.22), and disappointment regarding the true nature of professional practice (mean; 4.20). Academics reported that the COVID-19 pandemic moderately affected young people’s decision to pursue a career in nursing (mean; 3.18). Educational constraints were considered the least important factor, with a mean score of 2.23.

### 3.3. Association Between Demographic Characteristics and Contributing Factors

**Figure 1:**
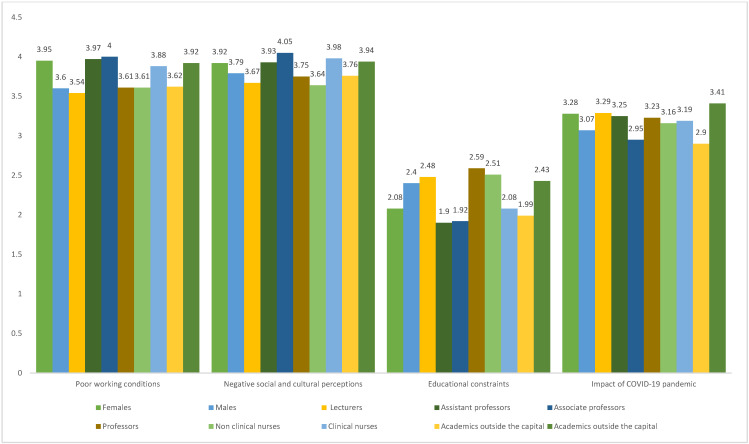
Academics’ views on factors contributing to the low interest in baccalaureate nursing education programs according to different demographic groups.

Table 3 and Figure 1 presents the association between demographic characteristics and academics’ views on factors contributing to the low interest in baccalaureate nursing education programs. Academics in nursing departments located in the capital considered the impact of the COVID-19 pandemic to be more significant compared to their counterparts working outside the capital (mean; 3.41 vs. 2.90, *p*-value = 0.037). Additionally, academics affiliated with nursing departments situated in the capital city perceived the impact of educational constraints on students’ career choices as more significant compared to their colleagues employed in institutions located outside the capital (mean; 2.43 vs. 1.99, *p*-value = 0.015). Academics without prior clinical nursing experience attributed greater importance to educational constraints as influencing factor (mean; 2.51 vs. 2.08, *p*-value = 0.023).

### 3.4. Motives

Academics identified the improvement in working conditions (mean; 4.82), increase in salaries (mean; 4.75), and the provision of scholarships and financial support for students (mean; 4.19) as the most influential incentives for attracting young individuals to baccalaureate nursing education (Table 4). Informative sessions on future career opportunities in research, education, and specialized nursing fields (mean; 4.19), along with the integration of advanced technology and innovative practices into the curriculum (mean; 3.99), were also identified as potential strategies to enhance the attractiveness of nursing programs among young individuals. Finally, information campaigns delivered through school visits, practical workshops, social media platforms, and traditional media outlets were also considered potential strategies for increasing the appeal of nursing programs among young individuals.

## 4. Discussion

This study examined the factors that discourage young individuals from enrolling in baccalaureate nursing education programs, as perceived by academic staff. Additionally, it explored potential incentives that could enhance the attractiveness of nursing education for prospective students. The findings indicate that academics identify several critical deterrents, including unfavorable working conditions, negative societal and cultural perceptions of the profession, educational limitations, and the impact of the COVID-19 pandemic. These factors collectively influence young people’s decision-making regarding a nursing career.

The inability of healthcare organizations’ leadership to ensure a healthy work environment that is supportive of nurses within the highly demanding context of nursing care has consistently been the most important factor driving nurses out of the profession. As nurses no longer perceive improvements in their working conditions, they increasingly consider leaving the profession. Studies conducted after the end of the pandemic, once the pressure on healthcare organizations has subsided, indicate that the proportion of nurses reporting an intention to leave the profession ranges from 50% to 70%, with the work environment emerging as a key determining factor [27,47,48,49]. Indeed, over time, the proportion of nurses who express an intention to leave the profession has increased substantially, representing a ticking time bomb for the very foundations of healthcare systems [50]. An important organizational factor associated with turnover intention is the salary of nursing staff. Thus, in combination with difficult working conditions and a non-supportive work environment, inadequate remuneration for nurses’ work creates a cumulative pattern of job dissatisfaction that ultimately facilitates their departure from the profession [51,52,53]. Enhancements in nurses’ working conditions and salaries were identified by respondents as two key motivating factors in the present study for the students to study nursing. The United Nations have included “Good Health and Well-Being” among the 17 Sustainable Development Goals, with one of the key prerequisites for achieving this goal being the increase in health financing and the recruitment, development, training, and retention of the health workforce in developing countries [54].

The public image of nurses and the recognition of their contribution may influence nurses’ decisions to leave the profession [55]. Although nurses were often portrayed as heroes during the COVID-19 pandemic, their image in society and in the media is still frequently shaped by stereotypes that depict them as predominantly female, low-skilled professionals with low social status, remuneration, academic qualifications and entry requirements, and limited professional autonomy [56]. According to nurses themselves, patients often lack awareness of the nursing role, frequently confusing nurses with other healthcare professionals. Also, while physicians are generally portrayed in a positive light, nurses are viewed less favorably, largely due to limited public knowledge of their qualifications and professional competencies [57]. The responsibility for improving the public image of nurses lies with nurses themselves, healthcare organizations, and policymakers [58].

Career counseling in schools regarding the nursing profession can strengthen students’ intentions to pursue nursing as a career [59]. Within the context of vocational guidance, it is essential to highlight the diverse professional opportunities available to nurses, which extend beyond hospital-based employment to include positions in primary healthcare, public health programs, research centers, and educational settings as school nurses. A highly unfavorable policy decision in the United States concerning the non-recognition of nursing as a “Professional Degree” may influence many students’ decisions to enter the nursing profession. This non-recognition is expected to reduce by 50% the amount of federal student loan funding available to nursing students. Such reduced access to this critical financial resource may serve as a significant deterrent to choosing nursing studies [60]. A common strategy adopted by many OECD countries to address their nursing workforce needs is the recruitment of foreign-trained nurses. Among OECD member states, the United Kingdom, Switzerland, New Zealand, and Australia exhibit the highest contribution of foreign-trained nurses to the growth of the nursing workforce [29].

The findings of the present study can be leveraged at multiple levels to strengthen the nursing profession. Given that students constitute the primary pipeline of the future nursing workforce, career guidance at the secondary education level can play a pivotal role. Informing students about the broad range of career opportunities available to nurses beyond hospital settings (e.g., research centers, academic careers, pharmaceutical companies), addressing potential misconceptions about the nursing profession and its public image (e.g., professional rights, participation in committees and senior organizational councils), and highlighting opportunities for postgraduate and doctoral education that provide specialized training associated with improved remuneration and career prospects are essential actions.

At the undergraduate level, universities can substantially enhance the nursing profession by upgrading curricula with a clear emphasis on clinical judgment, evidence-based practice, patient safety, quality, chronic care, and home-based/digital care while simultaneously developing specialization pathways and short certifications in critical competencies. Clinical education can also be strengthened through simulation, competency-based assessment, and structured mentorship/preceptorship programs in collaboration with clinical partners, ensuring that students acquire safe, measurable, and contemporary clinical experience. Furthermore, universities can bridge the education–labor market gap through career offices, alumni networks, transition (residency) programs, and permanent mechanisms of collaboration with hospitals and community services while concurrently promoting research and knowledge translation into practice through laboratories, student participation in projects, and the production of practice-oriented protocols and policy briefs that inform decision-making. Finally, they can enhance the prestige and social image of nursing through targeted outreach, campaigns, and events that present nurses as scientists and clinical leaders; invest in the development of educators and clinical partners (continuous professional development, joint academic–clinical appointments); and strengthen internationalization through exchanges and joint programs, thereby creating a coherent ecosystem that increases attractiveness, professional autonomy, and retention of nurses within the health system.

Ultimately, nurse retention depends on policymakers and healthcare organization leadership. For policymakers and healthcare administrators, the recommendations can be summarized as follows: legislating safe staffing ratios; establishing clear clinical career pathways (staff nurse–specialist–advanced practice–leadership) with corresponding salary recognition; funding transition programs for newly graduated nurses (residency/mentorship) and continuing education; strengthening professional autonomy; expanding nurse-led services (e.g., chronic disease management, prevention, home-based care); improving working conditions (flexible and fair scheduling, violence prevention, psychosocial support, adequate infrastructure); systematically reducing administrative burden through digitalization of documentation and supportive staff; adopting a safety culture and “just culture” with non-punitive incident reporting; and implementing a unified framework of indicators (burnout, turnover, vacancies, patient safety) to ensure data-driven decision-making and accountability.

This study has several limitations that should be acknowledged. First, although all academics from nursing departments in Greece were invited to participate, the response rate was 54.2%, which may introduce non-response bias and limit the representativeness of the sample. A recent meta-analysis found that the average response rate of online surveys such our study is 44.1% [61]. Response rates should be approximately 60% in cross-sectional studies like ours [62]. Although the response rate in our study covers the requirements, non-response bias is probable. For instance, the proportion of lecturers in our study was very low, and because they are among the most recently hired academic staff, their perspectives may differ from those of senior professors who have worked in nursing universities for many years. Second, data were collected using self-reported questionnaires, making this study susceptible to information bias. Third, the cross-sectional design precludes the establishment of causal relationships between demographic characteristics and academics’ views on factors contributing to low enrollment in baccalaureate nursing education programs. Furthermore, while the findings may reflect the Greek context, they cannot be generalized to other countries. Given that this is the first study to investigate factors influencing low enrollment in nursing programs at the university level, further research is warranted. Future studies should replicate this work in diverse international settings to enhance external validity. Additionally, exploring the perspectives of prospective students and parents could provide a more comprehensive understanding of the determinants of enrollment decisions. Longitudinal studies with larger and more representative samples are recommended to generate stronger evidence and capture temporal trends in attitudes and behaviors.

## 5. Conclusions

The shortage of nurses is a global phenomenon that jeopardizes the very foundations of healthcare systems. Nursing students constitute the primary pipeline supplying the nursing workforce. To our knowledge, this is the first international study to investigate academics’ perspectives on students’ reluctance to pursue nursing as a field of study. According to the participating academics, a poor nursing work environment, insufficient recognition of the nursing profession, inadequate career guidance, and the negative impact of the COVID-19 pandemic emerged as key deterrents to choosing nursing studies. Given the robust body of evidence on the factors driving nurses to leave the profession and discouraging students from entering it, timely decision-making at both policy and organizational levels regarding these issues is now crucial to reverse this trend and to secure an adequate supply of nurses.

## Figures and Tables

**Table 1:** Demographic characteristics of our respondents (n = 90).

Characteristics	N	%
Gender		
Females	47	52.2
Males	43	47.8
Age (years) ^a^	53.2	7.5
Rank		
Lecturers	14	15.6
Assistant professors	24	26.7
Associate professors	21	23.3
Professors	31	34.4
Work experience as academic (years) ^a^	12.4	8.9
Work experience as clinical nurse		
No	32	35.6
Yes	58	64.4
Academics in nursing departments located in the capital of the country		
No	40	44.4
Yes	50	55.6

^a^ mean, standard deviation.

**Table 2:** Factors contributing to the low interest in baccalaureate nursing education programs based on the perspectives of academic staff in nursing departments in Greece.

Factors	Mean	Standard Deviation	Median	Interquartile Range
Poor working conditions	3.79	0.83	4	0.8
Low salaries	4.27	1.05	5	1
Poor work environment	4.53	0.91	5	1
Professional responsibilities inherent in nursing practice	3.33	1.22	3	1
Bullying-mobbing experienced by nurses in the workplace	3.02	1.35	3	2
Negative social and cultural perceptions	3.86	0.89	4	1.3
Lack of social recognition	4.22	0.97	5	1
Limited career prospects	3.63	1.14	4	2
Disappointment regarding the true nature of professional practice	4.20	1.07	5	1
Psychological strain experienced by nurses as a result of the profession’s nature	3.69	1.10	4	2
Nursing profession’s limited social and professional prestige	3.55	1.33	4	2.5
Educational constraints	2.23	0.85	2	0.9
Insufficient academic preparation	1.89	1.19	1	1
Minimum academic threshold for university admission	2.53	1.32	2	1
Limited career counseling in schools	3.02	1.31	3	2
Former technological institutes transitioned into universities	1.64	1.04	1	1
Slow technological upgrading in universities	2.39	1.23	2	2
Transition of technological institutes into universities has contributed to students becoming less engaged with clinical aspects of nursing	1.91	1.23	1	2
Impact of the COVID-19 pandemic	3.18	1.15	3	1.5
Burden caused to nurses by the COVID-19 pandemic	3.31	1.21	3	1.5
Lack of recognition of the contribution of nurses in dealing with the COVID-19 pandemic	3.04	1.34	3	2

**Table 3:** Association between demographic characteristics and academics’ views on factors contributing to the low interest in baccalaureate nursing education programs.

Characteristics	Poor Working Conditions, Mean (SD)	*p*-Value	Negative Social and Cultural Perceptions, Mean (SD)	*p*-Value	Educational Constraints, Mean (SD)	*p*-Value	Impact of COVID-19 Pandemic, Mean (SD)	*p*-Value
Gender		0.054 ^a^		0.529 ^a^		0.082 ^a^		0.403 ^a^
Females	3.95 (0.83)		3.92 (0.91)		2.08 (0.77)		3.28 (1.20)	
Males	3.60 (0.81)		3.79 (0.87)		2.40 (0.92)		3.07 (1.09)	
Age	−0.21 ^b^	0.055 ^b^	−0.14 ^b^	0.177 ^b^	−0.05 ^b^	0.646 ^b^	−0.12 ^b^	0.255 ^b^
Rank		0.159 ^c^		0.547 ^c^		0.104 ^c^		0.784 ^c^
Lecturers	3.54 (1.29)		3.67 (1.19)		2.48 (1.18)		3.29 (1.31)	
Assistant professors	3.97 (0.60)		3.93 (0.71)		1.90 (0.45)		3.25 (1.19)	
Associate professors	4.00 (0.40)		4.05 (0.74)		1.92 (0.46)		2.95 (0.92)	
Professors	3.61 (0.92)		3.75 (0.96)		2.59 (0.98)		3.23 (1.22)	
Work experience as academic	−0.06 ^d^	0.608 ^d^	−0.01 ^d^	0.902 ^d^	0.15 ^d^	0.172 ^d^	0.01 ^d^	0.997 ^d^
Work experience as clinical nurse		0.186 ^a^		0.088 ^a^		0.023 ^a^		0.912 ^a^
No	3.61 (1.04)		3.64 (0.98)		2.51 (1.01)		3.16 (1.09)	
Yes	3.88 (0.69)		3.98 (0.82)		2.08 (0.73)		3.19 (1.19)	
Academics in nursing departments located in the capital of the country		0.086 ^a^		0.388 ^a^		0.015 ^a^		0.037 ^a^
No	3.62 (0.87)		3.76 (0.87)		1.99 (0.79)		2.90 (1.09)	
Yes	3.92 (0.78)		3.94 (0.91)		2.43 (0.86)		3.41 (1.16)	

^a^ independent samples t-test; ^b^ Pearson’s correlation coefficient; ^c^ analysis of variance; ^d^ Spearman’s correlation coefficient; SD: standard deviation.

**Table 4:** Motives for attracting young individuals to nursing programs, as perceived by academics in nursing departments in Greece.

Motives	Mean	Standard Deviation	Median	Interquartile Range
Salary improvement	4.75	0.66	5	0
Working conditions’ improvement	4.82	0.63	5	0
Scholarships and financial support for students	4.19	0.90	4	1
Incorporating advanced technology and innovative practices into the curriculum	3.99	1.03	4	2
Three-year study programs instead of four years	2.09	1.37	1	2
Information campaigns by nursing departments on social media	3.72	1.14	4	2
Information campaigns by nursing departments on traditional media	3.43	1.09	4	1
Engaging students in schools through school visits, presentations, and practical workshops	3.83	1.00	4	1.5
Organizing informative sessions on future career opportunities in nursing	3.72	1.14	4	2
Organizing informative sessions on future career opportunities in research, education, and specialized nursing fields	4.19	0.99	4	1

## Data Availability

The data presented in this study are available at: https://doi.org/10.6084/m9.figshare.30479528.v1 (accessed on 29 October 2025).

## Supplementary Materials

The following supporting information can be downloaded at: https://www.mdpi.com/article/10.3390/nursrep16020049/s1, Table S1: Factors contributing to the low interest in nursing university programs based on the perspectives of academic staff in nursing departments in Greece; Table S2: Motives for attracting young individuals to nursing programs, as perceived by academics.

## Public Involvement Statement

No public involvement in any aspect of this research.

## Guidelines and Standards Statement

This manuscript was drafted against the Strengthening the Reporting of Observational studies in Epidemiology (STROBE) guidelines for cross-sectional studies.

## Abbreviations

The following abbreviations are used in this manuscript:

PTSD	Post-traumatic stress disorder
PISA	Programme for International Student Assessment
STROBE	Strengthening the Reporting of Observational Studies in Epidemiology
SD	Standard deviation
