# Supplementary material for: Low Interest Among Young People in Becoming Nurses in Greece: Contributing Factors According to Academic Staff
Source: Nurs Rep. 2026 Jan 30;16(2):49. doi: 10.3390/nursrep16020049 (PMC12943630; doi:10.3390/nursrep16020049)
Supplement: Supplementary file 1 [file nursrep-16-00049-s001.zip › Table S2.pdf]

Table S2. Motives for attracting young individuals to nursing programs, as perceived by academics.

1. Salaries improvement
2. Working conditions improvement
3. Scholarships and financial support for students
4. Incorporating advanced technology and innovative practices into the curriculum
5. Three year study programs instead of four-years
6. Information campaigns by nursing departments on social media
7. Information campaigns by nursing departments on traditional media
8. Engaging students in schools through school visits, presentations, and practical workshops
9. Organizing informative sessions on future career opportunities in nursing
10. Organizing informative sessions on future career opportunities in research, education, and specialized nursing fields
