# Supplementary material for: Low Interest Among Young People in Becoming Nurses in Greece: Contributing Factors According to Academic Staff
Source: Nurs Rep. 2026 Jan 30;16(2):49. doi: 10.3390/nursrep16020049 (PMC12943630; doi:10.3390/nursrep16020049)
Supplement: Supplementary file 1 [file nursrep-16-00049-s001.zip › Table S1.pdf]

Table S1. Factors contributing to the low interest in baccalaureate nursing education programs based on the perspectives of academic staff in nursing departments in Greece.

**Poor working conditions**
1. Low salaries
2. Poor work environment
3. Professional responsibilities inherent in nursing practice
4. Bullying-mobbing experienced by nurses in the workplace
**Negative social and cultural perceptions**
5. Lack of social recognition
6. Limited career prospects
7. Disappointment regarding the true nature of professional practice
8. Psychological strain experienced by nurses as a result of the profession's nature
9. Nursing profession's limited social and professional prestige
**Educational constraints**
10. Insufficient academic preparation
11. Minimum academic threshold for university admission
12. Limited career counseling in schools
13. Former technological institutes transitioned into universities
14. Slow technological upgrading in universities
15. Transition of technological institutes into universities has contributed to students becoming less engaged with clinical aspects of nursing
**Impact of the COVID-19 pandemic**
16. Burden caused to nurses by the COVID-19 pandemic
17. Lack of recognition of the contribution of nurses in dealing with the COVID-19 pandemic
